# P-98. The Platform Trial In COVID-19 priming and BOOsting (PICOBOO): the immunogenicity, reactogenicity, and safety of different COVID-19 vaccinations administered as a second booster (fourth dose) in BNT162b2 primed individuals aged 18-< 50 and 50-< 70 years old

**DOI:** 10.1093/ofid/ofae631.305

**Published:** 2025-01-29

**Authors:** Charlie McLeod, Michael Dymock, Katie Flanagan, Magdalena Plebanski, Helen Marshall, Marie Estcourt, Ushma Wadia, Christian Tjiam, Christopher C Blyth, Kanta Subbarao, Francesca Mordant, Suellen Nicholson, Saul N Faust, Ruth Thornton, Anne McKenzie, Julie Marsh, Thomas L Snelling, Peter Richmond

**Affiliations:** Telethon Kids Institute, Perth, Western Australia, Australia; Telethon Kids Institute, Perth, Western Australia, Australia; Launceston General Hospital, Launceston, Tasmania, Australia; Royal Melbourne Institute of Technology University (RMIT), Melbourne, Victoria, Australia; University of Adelaide, Adelaide, South Australia, Australia; University of Sydney, Sydney, New South Wales, Australia; Telethon Kids Institute, Perth, Western Australia, Australia; Telethon Kids Institute, Perth, Western Australia, Australia; Wesfarmers Centre of Vaccines and Infectious Diseases, Telethon Kids Institute, Nedlands, Western Australia, Australia; WHO Collaborating Centre for Reference and Research on Influenza, Melbourne, Australia, Melbourne, Victoria, Australia; Doherty Institute, Melbourne, Victoria, Australia; University of Melbourne, Melbourne, Victoria, Australia; University of Southampton and University Hospital Southampton NHS Foundation Trust , Southampton, England, United Kingdom; Telethon Kids Institute, Perth, Western Australia, Australia; Telethon Kids Institute, Perth, Western Australia, Australia; Telethon Kids Institute, Perth, Western Australia, Australia; University of Sydney, Sydney, New South Wales, Australia; University of Western Australia School of Medicine, Perth’s Children Hospital, Nedlands, Western Australia, Australia

## Abstract

**Background:**

PICOBOO is a randomised, adaptive trial evaluating the immunogenicity, reactogenicity, and safety of COVID-19 booster strategies. Here, we report data for second boosters among individuals aged 18-< 50 and 50-< 70 years old primed with BNT162b2 (18-< 50y-BNT162b2 and 50-< 70y-BNT162b2, respectively) until Day (D) 84.

CONSORT diagram for participants recruited to the 18-<50y-BNT162b2 (A) and 50-<70y-BNT162b2 (B) strata for second booster vaccines. Participants were excluded from subsequent analyses if they were infected with COVID-19 (C19 Infection) or had withdrawn. Participants that missed visits (Missed) were eligible to be included in subsequent analyses.

**Figure:**
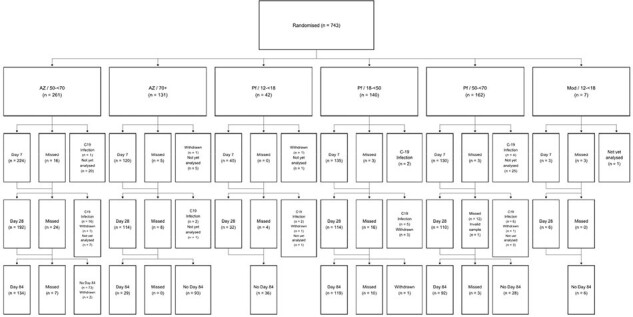

CONSORT diagram for participants recruited to the 18-<50y-BNT162b2 (A) and 50-<70y-BNT162b2 (B) strata for second booster vaccines. Participants were excluded from subsequent analyses if they were infected with COVID-19 (C19 Infection) or had withdrawn. Participants that missed visits (Missed) were eligible to be included in subsequent analyses.

**Methods:**

Immunocompetent adults who received any licensed first booster at least three months prior were eligible. Participants were randomly allocated to BNT162b2, mRNA-1273 or NVX-CoV2373 1:1:1. The log_10_ concentration of anti-spike IgG was summarised as the geometric mean concentration (GMC). Reactogenicity and safety outcomes were captured. Additional analyses were performed on a subset. ACTRN12622000238774.

Baseline characteristics for study participants recruited to the 18-<50y-BNT162b2 and 50-<70y BNT162b2 strata for second booster vaccines summarised according to study arm.

**Figure:**
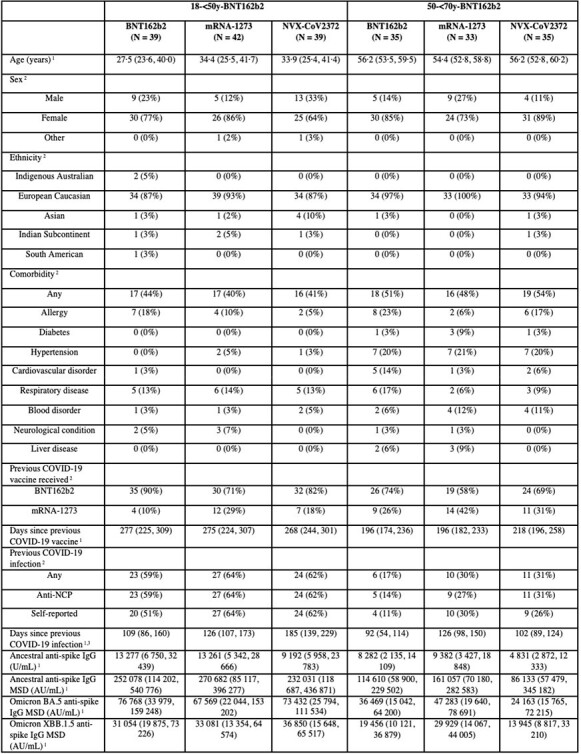

Baseline characteristics for study participants recruited to the 18-<50y-BNT162b2 and 50-<70y BNT162b2 strata for second booster vaccines summarised according to study arm.

**Results:**

Between 29 Mar 2022 and 7 Jul 2023, 743 participants were recruited to the platform with D28 samples; 120 belonged to the 18-< 50y-BNT162b2 and 103 belonged to the 50-< 70y-BNT162b2 strata. At D28, the GMCs (95% credible intervals) were 40 338 (32 978, 47 813), 48 117 (39 603, 57 826) and 24 220 (19 586, 29453) U/L in the 18-< 50y-BNT162b2 stratum following BNT162b2, mRNA-1273 and NVX-CoV2372, respectively. Corresponding values in the 50-< 70y-BNT162b2 stratum were 29 344 (23 763, 35 163), 36 026 (29 042, 43456) and 16 040 (12 923, 19 510) U/L. By D84, GMCs fell to 24 643 (17 779, 31 708), 33 236 (24 078, 42, 798) and 20 898 (14 978, 27 596) in the 18-< 50y-BNT162b2 stratum, respectively. D84 GMCs in the 50-< 70y-BNT162b2 stratum were 19 782 (14 157, 25 771), 24 817 (17 740, 32 024) and 15 310 (11 0349, 19 784) U/mL. At D28, neutralisation against wild-type virus was 172, 211 and 74 IU/mL following BNT162b2, mRNA-1273 and NVX-CoV2372. By D84, this fell to 95, 157 and 72 IU/mL. Limited neutralisation against BA.5 and XBB1.5 was found following all vaccines. Severe reactogenicity events were low (< 5%). Additional data will be available at the time of presentation.

Posterior distributions of the anti-spike IgG adjusted GMC against Ancestral SARS-CoV-2 at D7, D28 and D84 for each study arm in participants recruited to the 18-<50y-BNT162b2 and 50-<70y-BNT162b2 strata for second booster vaccines without COVID-19 infection after randomisation and before D28 (D7 for D7 distributions).

**Figure:**
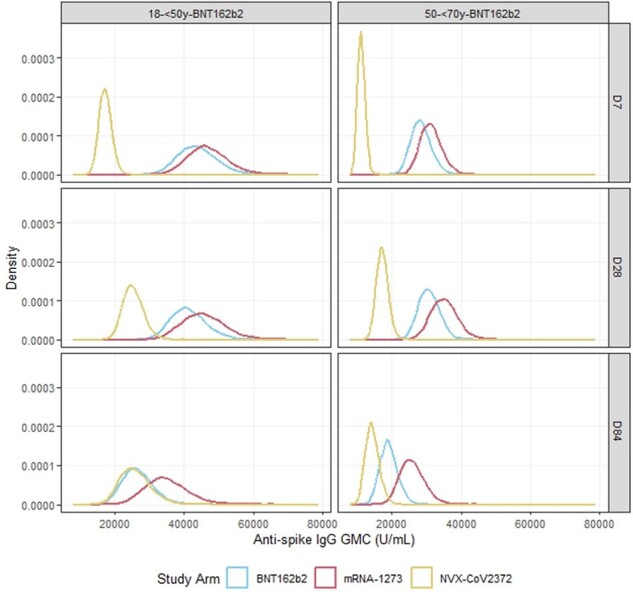

Posterior distributions of the anti-spike IgG adjusted GMC against Ancestral SARS-CoV-2 at D7, D28 and D84 for each study arm in participants recruited to the 18-<50y-BNT162b2 and 50-<70y-BNT162b2 strata for second booster vaccines without COVID-19 infection after randomisation and before D28 (D7 for D7 distributions).

**Conclusion:**

Each COVID-19 vaccine elicited boosted antibody responses to wild-type virus among BNT162b2-primed adults. GMCs and neutralisation titres were higher in the younger cohort compared to older adults. Minimal neutralisation was observed against Omicron subvariants, highlighting the need for boosting with vaccines with greater specificity for Omicron subvariants.

Posterior distributions of the adjusted geometric mean NF50 of Ancestral SARS-CoV-2 at D28 and D84 for each study arm in participants recruited to the 18-<50y-BNT162b2 and 50-<70y-BNT162b2 strata for second booster vaccines without COVID-19 infection after randomisation and before D28 in the immunological subset.

**Figure:**
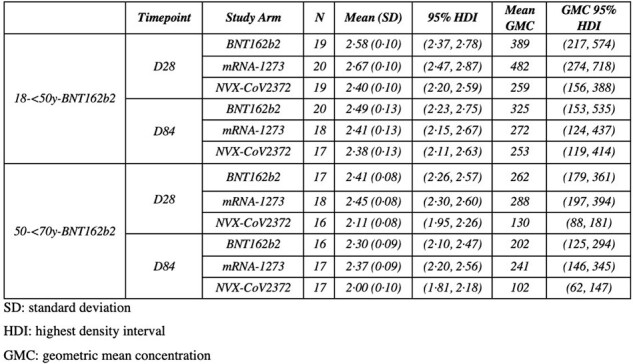

Posterior distributions of the adjusted geometric mean NF50 of Ancestral SARS-CoV-2 at D28 and D84 for each study arm in participants recruited to the 18-<50y-BNT162b2 and 50-<70y-BNT162b2 strata for second booster vaccines without COVID-19 infection after randomisation and before D28 in the immunological subset.

**Disclosures:**

**Magdalena Plebanski, PhD**, AstraZeneca: Grant/Research Support **Helen Marshall, MD**, ILiAD biotechnologies: Grant/Research Support **Saul N. Faust, FRCPCH PhD**, AstraZeneca: Grant/Research Support|BioNTech: Grant/Research Support|GSK: Grant/Research Support|Iliad Biotechnologies: Grant/Research Support|J&J: Grant/Research Support|J&J: Advisor, no personal payments (all honoraria paid to employing hospital)|Moderna: Grant/Research Support|Novavax: Advisor, no personal payments (all honoraria paid to employing hospital)|Pfizer: Advisor, no personal payments (all honoraria paid to employing hospital)|Sanofi: Grant/Research Support|Sanofi: Advisor, no personal payments (all honoraria paid to employing hospital)|Valneva: Grant/Research Support **Peter Richmond, MBBS**, ILiAD biotechnologies: Grant/Research Support

